# Duration of respiratory sample stability at -80ºC for SARS-CoV-2 PCR

**DOI:** 10.12669/pjms.38.ICON-2022.5777

**Published:** 2022-01

**Authors:** Javeria Aijaz, Fouzia Naseer, Maqboola Dojki, Saba Jamal

**Affiliations:** 1 Javeria Aijaz, FCPS. PhD, Pathology Department, Indus Hospital & Health Network Karachi, Pakistan; 2 Fouzia Naseer, MSc, MBA, Pathology Department, Indus Hospital & Health Network Karachi, Pakistan; 3 Maqboola Dojki, BS (MT ASCP), Pathology Department, Indus Hospital & Health Network Karachi, Pakistan; 4 Saba Jamal, Diplomat American Board of Anatomic and Clinical Pathology, Pathology Department, Indus Hospital & Health Network Karachi, Pakistan

**Keywords:** COVID-19, SARS-CoV-2, PCR, specimen retention, respiratory, ultra-freezer, biorepository

## Abstract

**Objective:**

To determine the stability of respiratory samples for SARS-CoV-2 PCR at standard laboratory ultra-freezer temperatures (-80°C).

**Methods:**

Five hundred and sixty-five archived, SARS-CoV-2 PCR positive patient specimens received at the Pathology Department of the Indus Hospital & Health Network between January 2021 and June 2021 were retested in June 2021. Samples had been stored at -70°C or below throughout this duration. Sample integrity following storage was assessed as the percentage of samples with reproducible results, and as consistency of cycle threshold (Ct) values between the original testing and the repeat testing.

**Results:**

Of the 565 samples evaluated in this study, 86% gave reproducible results upon retesting. However, there was no correlation between the duration of storage and result reproducibility, though the majority (69% for PCR Target-I and 78% for PCR Target-II respectively) of non-reproducible results had Ct values above 30. Similarly, there was a consistent increase of Ct values upon storage at ultra-freezer temperatures, though the effect again was more contingent upon freezing the sample in the ultra-freezer rather than the duration of storage.

**Conclusion:**

SARS-CoV-2 positive respiratory specimens for PCR can be stored for up to six months at -70°C or below without loss of sample integrity, though there is some loss of PCR-detected viral targets as evidenced by an immediate increased in the PCR-generated Ct values. In addition, samples with initial Ct values above 30 are more likely to give non-reproducible results.

## INTRODUCTION

Clinical laboratories are mandated by guidelines of accrediting agencies (CLIA, CAP, TJC) to have plans in place for patient specimen retention and storage. Such retention serves several purposes including repeat or additional testing when needed, further investigation for public health purposes, quality control procedures, new test validation, and subsequent research if needed.^1^Specimen storage conditions, including appropriate temperatures and durations, need to be defined in order to ensure that specimens remain uncompromised throughout the retention period, and any subsequent analyses on the specimens generate valid results.

While specimen storage in clinical laboratories is desirable, it is nevertheless resource-intensive, including the requirement for uninterrupted power supply and storage space in freezers and refrigerators. A specimen storage plan should thus ideally balance wastage of this precious resource against wastage of laboratory resources required to store specimens. This necessitates the systematic testing of the upper time limits of storage at standard storage temperatures of laboratory refrigerators and freezers, as storage beyond these temperatures would involve use of resources without any benefit to the clinical laboratory.

CDC guidelines recommend storage of respiratory specimens at 2-8°C for up to 72 hours after collection, while if a delay in testing or shipping is expected, such specimens are recommended to be stored at -70°C or below.^2^,^3^ This is a general recommendation, however, for all respiratory samples, and not specifically for samples to be tested for SARS-CoV-2 through PCR. In addition, the recommendation does not include an upper time limit for storage at -70°C that can safely be undertaken without compromising sample integrity. One FDA recommendation states that respiratory samples can be stored at -70°C or below for up to 8 weeks without any deterioration.^4^ It is unclear, however, if respiratory specimens remain viable beyond this time.

With reference to SARS-CoV-2 detection through PCR in respiratory specimens specifically, there are several studies aimed at evaluating sample integrity following short-term storage at room temperature and at refrigerator temperatures (2-8°C).^5^-^9^ There is, however, a paucity of studies evaluating sample viability upon long-term storage at standard ultra-freezer temperatures (-70 to -80°C). Guidelines for the SARS-CoV-2 PCR kits used in this study also state that respiratory specimen stability at standard refrigerator and freezer temperatures has not been established.^10^ Moreover, in the current pandemic, these samples may especially be needed for research purposes, for example, to study the evolution of the virus and its mutation pattern. However, since such storage is not without cost to a clinical laboratory, there is a need to determine the duration for which samples remain viable at the standard ultra-freezer temperatures (below -70°C) employed for bio-repositories associated with clinical laboratories. The primary aim of this study was thus to determine the duration for which samples for SARS-CoV-2 PCR remain viable at standard ultra-freezer temperatures in clinical laboratories.

## METHODS

Five hundred and sixty-five randomly selected, anonymous, archived, SARS-CoV-2 PCR positive nasopharyngeal patient specimens stored in viral transport, media were used in this study. These specimens had been sent to the Pathology Department at Indus Hospital & Health Network, Karachi, after approval by the Institutional Review Board (IHHN_IRB_2021_07_001) for routine testing for SARS-CoV-2 between January 2021 and June 2021. Samples had been stored at 4°C from the time of collection to the time of testing, in accordance with standard recommendations. Storage time at 4°C before the initial testing ranged between two hours and 24 hours. After PCR testing, the remnant samples were stored at -80°C until their evaluation for this study.

Real-time PCR testing on these samples was done either on Roche Cobas 6800 using cobas SARS-CoV-2 kit or on GeneXpert using Xpert® Xpress SARS-CoV-2. Both systems are fully automated, including sample preparation (nucleic acid extraction and purification) followed by PCR amplification and detection systems. In addition, both systems detect two targets in the virus genome – a ‘Target-I’ which is a nucleic acid sequence specific to SARS-CoV-2, and a ‘Target-II’ which is a nucleic acid sequence not specific to SARS-CoV-2, but is common to all Sarbecoviruses.

Samples selected for the study were re-run in June 2021. Thus, the storage period of these samples at -70 to -80°C ranged two prepositions between one and six months. Xpert® Xpress SARS-CoV-2 was used for the repeat run on all samples. None of the samples were thawed in between the first and the repeat runs, while the storage temperature was also monitored as part of the routine laboratory procedures to ensure that there is no significant deviation from the recommended storage temperatures of ultra-freezers. Table-I gives the number of samples selected from each month for inclusion in the study. Reproducibility of positive results and cycle threshold (Ct) values of samples at the time of the initial run, in comparison with those of the repeat run were used to evaluate sample integrity. Cycle threshold (Ct) is a numerical value generated during a RT-PCR test. It refers to the number of cycles needed for a sample to amplify and cross a threshold (cut-off) to be considered detected/positive.^11^ In addition, Ct values of samples, in general, are inversely correlated with pathogen load, infectivity, and severity of disease, as a lower Ct value means a higher amount of the target.^12^,^13^ Ct values at the time of testing were computed from already existing laboratory records, while Ct values after storage of samples were recorded after the second run on each sample undertaken for the purpose of this study. All values were computed using MS Excel, which was also used for data analysis.

**Table I:** Reproducibility of positive results on repeat testing.

	Jan-21	Feb-21	Mar-21	Apr-21	May-21	Jun-21
No. of samples	84	100	81	100	100	100
% Reproducibility	99	92	70	93	82	80

## RESULTS

The overall percentage reproducibility of SARS-CoV-2 PCR results was 86%. Table-I shows the reproducibility of results from the month-wise time points included in the study.

**Fig.1:**
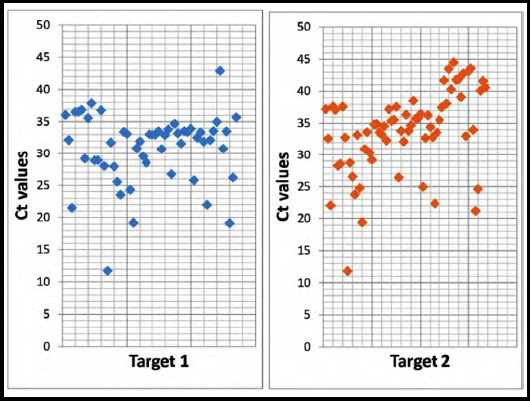
Distribution of initial Target-I and Target-II CT values of discrepant results i.e., positive results which turned negative on repeat testing.

Overall, 69% and 78% of discrepant results, i.e. positive results which turned negative on repeat testing, had a Ct value above 30 for Target-I and Target-II respectively. Fig.1 shows the distribution of initial Ct values obtained with discrepant positive results.

The average Ct values obtained for Target-I and Target-II during the initial run and subsequent run, along with the standard deviation and coefficient of variation are presented in Table-II.

**Table II:** Month-wiseean, SD, and CV of sample Ct values at collection and following storage.

Six months	Target-I-Original run (Jan-21)	Target-I- Re-run (Jun-21)	Target-II-Original run (Jan-21)	Target-II- Re-run (Jun-21)
Mean	28.96	29.87	31.00	32.00
Standard deviation	4.36	6.03	5.14	6.96
Coefficient of variation	15.07	20.19	16.59	21.76

*Five months*	*Target-I-Original run (Feb-21)*	*Target-I- Re-run (Jun-21)*	*Target-II-Original run (Feb-21)*	*Target-II- Re-run (Jun-21)*

Mean	28.42	30.12	29.11	31.98
Standard deviation	5.17	6.30	5.79	6.52
Coefficient of variation	18.18	20.91	19.89	20.38

*Four months*	*Target-I-Original run (Mar-21)*	*Target-I- Re-run (Jun-21)*	*Target-II-Original run (Mar-21)*	*Target-II- Re-run (Jun-21)*

Mean	29.63	31.37	31.28	33.77
Standard deviation	3.74	5.98	5.68	6.17
Coefficient of variation	12.52	19.08	18.15	18.26

*Three months*	*Target-I-Original run (Apr-21)*	*Target-I- Re-run (Jun-21)*	*Target-II-Original run (Apr-21)*	*Target-II- Re-run (Jun-21)*

Mean	27.62	29.17	28.68	31.73
Standard deviation	4.65	6.80	5.44	6.91
Coefficient of variation	16.83	23.31	18.95	21.77

*Two months*	*Target-I-Original run (May-21)*	*Target-I- Re-run (Jun-21)*	*Target-II-Original run (May-21)*	*Target-II- Re-run (Jun-21)*

Mean	27.55	30.99	28.70	33.08
Standard deviation	4.64	6.63	5.55	6.71
Coefficient of variation	16.85	21.41	19.34	20.27

*1 month or less*	*Target-I-Original run (Jun-21)*	*Target-I- Re-run (Jun-21)*	*Target-II-Original run (Jun-21)*	*Target-II- Re-run (Jun-21)*

Mean	28.39	30.94	32.34	32.73
Standard deviation	9.06	7.24	7.06	6.82
Coefficient of variation	31.90	23.39	21.84	20.85

**Fig.2:**
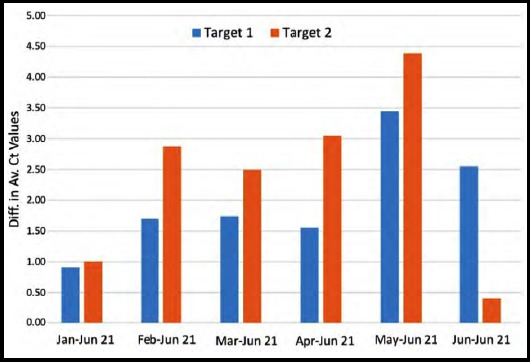
Difference in average CT values between the first run and the repeat run of samples of SARS-CoV2 PCR.

From the above data, it is evident that there is a consistent increase of Ct values targets upon retesting after storage at ultra-freezer temperatures. The average increase of Ct values for each of the targets across the month-wise time points is depicted in Fig.2.

**Fig.3:**
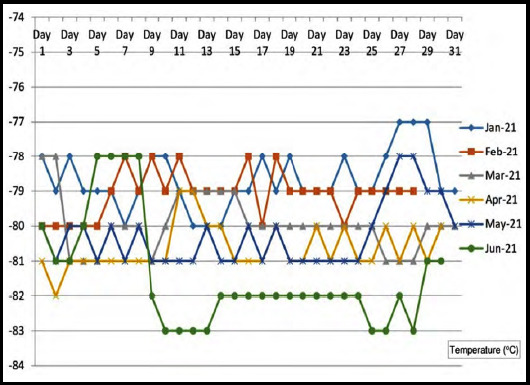
Temperature log of the ultra-freezer used of the study sample storage.

The temperature chart of the freezer used to store samples was maintained throughout at the recommended temperature of -70°C or below. All critical equipment at the study site is backed up with alternate power supplies. In addition, ultra-freezers are equipped with alarm systems in case their temperatures cross unacceptable limits. No untoward incident related to ultra-freezer use to store samples was reported in the study period. Fig.3 depicts the daily monitored temperature log of the ultra-freezer in which samples for this study were stored.

## DISCUSSION

Overall, the results of our study suggest that samples positive for SARS-CoV-2 through PCR remain viable at -70°C or below for at least up to six months. There are, however, two caveats in this general conclusion. Firstly, 14% percent of the initially positive samples turned out to be negative on re-evaluation. It must be noted, however, that most of these samples had a Ct value of 30 or above on initial testing. Ct values above 30 obtained from SARS-CoV-2 PCRs have been shown to correlate with samples containing non-viable virus. This may explain why discrepant results were mostly obtained with samples exhibiting Ct values above 30^14^. Notably, though there is a trend towards higher Ct values in discrepant results, no correlation was found for the percentage of discrepant results with sample storage duration. If anything, the samples stored for the longest period of time analyzed in the study – samples from January 2021 – showed the least number of discrepant results.

Secondly, there is a trend towards increased Ct values with sample storage, even in ultra-freezers. An increase in Ct value by 3.3 indicates approximately a 10-fold decrease in target amount, indicating that even storage at the standard laboratory ultra-freezer temperatures fails to prevent some loss of SARS-CoV-2 target constituents for PCR^15^. It must be noted that this loss starts immediately after storage, evidenced by the observation that Ct values increased even for samples with the shortest storage duration of less than a month, regardless of the duration of storage. However, the exact Ct values notwithstanding, samples generally found positive upon initial testing would generally again give a positive result.

Most existing guidelines on the appropriate storage of samples for SARS-CoV-2 PCR recommend storage of respiratory specimens at 2-8°C for up to 72 hours after collection. While it is clear that if a delay beyond 72 hours is anticipated, samples should be stored in ultra-freezers with temperatures of -70°C or below. There nevertheless remains a gap in defining the upper time limit of such storage, particularly with reference to SARS-CoV-2. Thus, there have been several studies evaluating SARS-CoV-2 PCR sample integrity over refrigerator and room temperatures,^5^-^9^ we have not come across any published, publicly available, study or data with which we could perform a direct comparison of the results obtained in this study.

### Limitations of the Study:

While this study has provided evidence to fill an important knowledge gap pertaining to SARS-CoV-2 sample storage in ultra-freezers, it has certain limitations which are as follows: Firstly, this study has not evaluated sample viability beyond 6 months of storage at temperatures tested in this study. This could not be accomplished due to the limitation in the resources available to the laboratory, particularly the kits available for PCR testing. It could have been possible to reduce the number of samples for each time period and to include more samples from prior time points, but this would have reduced the confidence in our results obtained.

Furthermore, the same specimen was not used for repeat testing, which would have shown a more standardized month-wise progression of Ct values. This could not be accomplished for practical reasons since actual remnant patient samples were used for this study. These would not have provided sufficient material for the 6 successive data points needed for this study.

Another limitation of the study was that it included only the initially positive samples, while negative samples were excluded from the study. This was done considering a resource-limitation regarding the number of tests which could have been performed for this study. Inclusion of negative samples would have divided the sample, the sample size for each analysis by half and hence reduced confidence in our results. Additionally, it is more practicaly significant to generate such data for positive samples, since these are more likely to be needed for any subsequent analyses. We also do not see any reason why the applicable results of initially positive samples cannot be extrapolated to the initially negative ones.

Lastly, although both of the PCR systems used for this study (Roche Cobas 6800 using cobas SARS-CoV-2 kit or on GeneXpert using Xpert® Xpress SARS-CoV-2) were fully automated and sensitive systems, use of a single system for the study would have increased the precision of results. Again, as the clinical laboratory uses multiple systems, and actual patient specimens were used for this study, the limitation could not be avoided for the purpose of this study.

## CONCLUSION

Initially, positive samples for SARS-CoV-2 PCR retain their integrity for up to six months following storage at standard laboratory ultra-freezer temperatures of -70°C or below. However, samples with a Ct value above 30 are more likely to give discrepant results after storage at these temperatures, though the likelihood of discrepant results appears to be unrelated to the duration of storage. In addition, there appears to be some loss of viral targets detected by real time PCR upon storage, as evidenced by a consistent increase of Ct values with storage. Interestingly, this increase of Ct values happens soon after storage and appears to be unrelated to the duration of storage within a timeframe of 6 months.

### Author`s Contribution:

**JA and MD** Conceived and designed the study.

**JA** Wrote the manuscript and is responsible for the integrity and accuracy of this study.

**FN** Did data collection, statistical analysis and prepared the figures.

**SJ** Did the editing, final review and approval of manuscript.
